# A grounded theory study on work related stress in professionals who provide health & social care for people who exhibit behaviours that challenge

**DOI:** 10.1371/journal.pone.0229706

**Published:** 2020-02-27

**Authors:** Daniel Rippon, Andrew McDonnell, Michael Smith, Michael McCreadie, Mark Wetherell

**Affiliations:** 1 University of Northumbria at Newcastle, Newcastle upon Tyne, United Kingdom; 2 Studio III Training Systems and Clinical Support, Warwick, United Kingdom; Tabriz University of Medical Sciences, IR Iran, ISLAMIC REPUBLIC OF IRAN

## Abstract

Providing direct health and social care services for people who exhibit behaviours that challenge can be a highly stressful occupation. Existing literature has suggested that there is a need to develop further theoretical understanding of how work related stress can be reduced in professions that consist of providing care for people who exhibit behaviours that challenge. The aim for this study was to use a Classic Grounded Theory approach to develop a theoretical framework to illustrate a common issue that could influence work related stress levels experienced when managing behaviours that challenge in health and social care settings. A series of focus groups and 1:1 semi-structured interviews were conducted to explore the articulated experiences of 47 health/social care professionals who provide care for people who exhibit behaviours that challenge. This led to the development of Therapeutic Engagement Stress Theory (TEST), which illustrates that the perceived capacity to therapeutically engage with people who exhibit behaviours that challenge is an issue that can influence the levels of stress experienced by health/social care professionals. TEST provides a framework that could be applied to identify specific factors that inhibit staff to successfully deliver caring interventions for people who exhibit behaviours that challenge, and also inform bespoke support mechanisms to reduce stress in health/social care professionals.

## Introduction

Behaviours that challenge have been defined as any behaviour of such intensity, frequency or duration as to compromise the wellbeing of the individual exhibiting the behaviour, or others, which can lead to the implementation of restrictive/aversive interventions or exclusion from community settings [1]. Providing care for people who exhibit behaviours that challenge has been identified as a prominent occupational hazard that can impact the levels of work related stress experienced by health and social care professionals [2]. Work related stress has been described as the harmful response to excessive pressures and demands that professionals experience as a result of their occupation [3]. Between 2014 and 2017, occupations associated with the delivery of health and social care had the greatest incidences of absenteeism due to work related stress in the UK [3]. Work related stress was also the most commonly reported reason for healthcare professionals, such as nurses, to consider leaving their profession [4]. It has also been observed that alongside people who work in protective services, such as policing, professionals who work within health/social care services encounter the most incidences of violence at work in comparison to employees who work in other industry sectors in the UK [5]. It is therefore necessary to gain an understanding on how health/social care professionals can be supported in their occupations, when providing care for people who exhibit behaviours that challenge, as a means to ensure employee wellbeing and reduce work related stress.

The onset of chronic work related stress in caring professions can be a precursor for occupational burnout and low job satisfaction [6]. It has been suggested that work related stress can contribute to the high turnover of staff observed in caring professions [7]. Newly qualified nurses have cited that providing care for patients who exhibit behaviours that challenge can be the most stressful aspect of their occupation, which can then elicit intentions to leave the nursing profession [8]. This would suggest that interventions that focus on reducing the work related stress, which can coincide with providing care for people who exhibit behaviours that challenge, may help in retaining staff and their expertise within caring professions. Thus, there is an essential requirement to gain further understanding of protective factors that may be conducive in negating work related stress and supporting frontline staff in the provision of care for people who exhibit behaviours that challenge.

Frontline health/social care professionals can also encounter a wide range of distressed behaviours, as exhibited by care recipients, which can create challenging situations in the workplace. Exposure to both verbal and physical aggression can contribute towards the work related stress experienced by health/social care professionals who work within older adult’s inpatient services [2]. Regular exposure to other behavioural symptoms such as apathy to engage in treatment interventions [9] and screaming [10], can also be detrimental to the wellbeing of health/social care professionals. Self-injurious behaviours exhibited by children with autism [11]; repetitive vocalisations in adults with an intellectual disability [12]; and impulsivity in people with a diagnosis of bipolar disorder [13], have also been identified as behavioural symptoms that could elicit challenging situations in the workplace for health/social care professionals. It has been posited that under stressful working conditions, healthcare professionals may also inadvertently trigger challenging incidences [14], particularly when care recipients concerned are experiencing impaired cognition [15]. However, it has been suggested that there are no significant direct relationships between work related stress and the type or frequency of behaviours that challenge [16]. This would suggest that there may be other work related factors that influence stress levels experienced by health/social care professionals who provide care for people who exhibit behaviours that challenge. Therefore, there is a need to ascertain a holistic understanding of the work related processes that may influence the stress levels experienced by frontline health/social care staff who are required to prevent or de-escalate incidences of behaviours that challenge in their occupation.

There are several existing theories that provide propositions as to how stress can occur within workplace settings. The Person-Environment Fit model [17] purports that work related stress can occur when employees perceive a discrepancy between personal needs and the way in which the working environment fulfils employee needs. The Demand-Control-Support model [18] suggests that the amount of stress experienced is dependent upon levels of work related demands, perceived level of control to complete tasks and the amount of support that is available to employees in their profession. According to Equity Theory [19], work related stress can occur when employees perceive that they provide more professional input into their interpersonal relationships and employing organisation than the amount of rewards received. The Transactional Model of Stress and Coping [20] suggests that work stress levels are dependent upon internal cognitive processes and the way in which employees cognitively appraise work related challenges. However, existing work stress theories have not been developed specifically to explain work related stress within professions that consist of providing health and social care to people who exhibit behaviours that challenge [21]. It has been suggested that stress models, which are occupation specific, may provide more relevant explanations on how to ameliorate stressors that are idiosyncratic to a particular profession [22]. Thus, there is a need to develop a theoretical framework that explains the specific conditions under which stress can occur, and also be reduced, in occupations that consists of the idiosyncratic demand of providing care for people exhibiting behaviours that challenge. Furthermore, existing work stress theories illustrate how stress can manifest [21], and thus there is a need to gain a greater theoretical understanding on how stressors can be reduced in professions where staff provide care for people who exhibit distress [23]. Therefore, the current study aimed to develop a theoretical framework that illustrated both the causes of and protective factors against occupational stress, which could be applied to inform bespoke strategies to support professionals who provide health and social care for people who exhibit behaviours that challenge.

## Method

### Design approach

A Classic Grounded Theory [24] methodology was utilised to investigate the causes of and protective factors against work related stress in health/social care professionals who work with care recipients who exhibit behaviours that challenge. Classic Grounded Theory (CGT) is a methodology of analysing data as a means to develop a theoretical framework that illustrates, explains and proposes methods of how people resolve a particular common issue that is specific to a social phenomenon of interest. Thus, the current study aimed to develop a theoretical framework to illustrate a common issue that could impact the levels of work related stress experienced by health and social care professionals who provide services for people who exhibit behaviours that challenge.

### Participants

A theoretical sampling strategy was employed in accordance with data collection protocols for conducting a CGT study. Theoretical sampling is a procedure that consists of the ongoing collection/analysis of data, coding of transcripts and reflection to inform who to recruit subsequently as a means to develop theoretical categories that illustrate the core concerns of the participants attached to the social phenomenon being investigated [25]. As part of the CGT methodology, it is recommended that a purposive sampling strategy is initially employed before commencing with the theoretical sampling process and refining the emerging theory [26]. It was therefore necessary to begin the study by recruiting a purposive sample (Table 1) of health/social care professionals as a means to ascertain the theoretical categories, or work related factors, that could provide possible explanations on how stress manifests in professions that concern the safe prevention/de-escalation of behaviours that challenge. Once this was completed, theoretical sampling commenced in order to refine the categories and verify the central problem (core category) that could explain work related stress specifically for professionals who encounter behaviours that challenge in the workplace. An integral inclusion criterion for the purposive and theoretical sample was that participants were required to provide health or social care duties for people who exhibit behaviours that challenge in a professional capacity at the time of data collection.

**Table 1:** Demographics and methods of data collection for the purposive sample.

Service	Method of Data Collection	Gender	Age	Occupation	Months of experience in stipulated role
Community Mental Healthcare Team	Focus Group	6 Female 4 Male	Range 31–64 years Mean = 47.00 years, SD = 11.45	1 Service Manager 1 Assistant Service Manager 3 Senior Support Workers 3 Support Workers 2 Housing Support Workers	Range 3–156 months Mean = 69.30 months, SD = 58.81
Children and Younger Person’s (CYPS) Mental Health Inpatient	1:1 Semi-structured interview	1 Female	53 years	Staff Nurse	45 months
Learning Disabilities Inpatient	1:1 Semi-structured interview	1 Female	27 years	Staff Nurse	21 months
CYPS Mental Health Inpatient setting	1:1 Semi-structured interview	1 Female	20 years	Support Worker	20 months

#### Strategy for recruitment

In order to gain opportunities for recruiting participants, the professional networks of authors DR, AM and MM, comprising of current/former colleagues at organisations that provide treatments and assessments for people who exhibit behaviours that challenge, were initially contacted by email. A request to recruit participants advert was also distributed through the professional networks of authors AM and MM. The aims of this study were then presented by author DR at relevant board meetings within organisations that expressed an interest for their employees to take part in this study. Potential participants were then contacted by line managers within the organisations that agreed to take part in the study. Author DR liaised with the relevant line managers as a means to arrange convenient dates and times to conduct the 1:1 interviews/focus groups with the participants who took part in the study.

#### Purposive sample

The purposive sample comprised of the following participants and the method in which the data were collected is presented in chronological order within Table 1. Data collection for the purposive sample took place between April 2016 and June 2016.

#### Theoretical sample

The purposive sampling stage served to identify appropriate professional groups who experience the demands of providing care for people who exhibit behaviours that challenge, across various specialism and settings, which informed the recruitment strategy for the theoretical sample. The theoretical sampling strategy is presented in chronological order within Table 2. Data collection for the theoretical sample took place between July 2016 and January 2017.

**Table 2:** Demographics and methods of data collection for the theoretical sample.

Service	Method of Data Collection	Gender	Age	Occupation	Months of experience in stipulated role
Community & Residential Autism Service	Focus Group	4 Females	Range 22–63 years Mean = 45.00 years, SD = 16.99	1 Senior Support Worker 3 Support Workers	Range 10–168 months Mean = 111.25 months, SD = 74.33
Community & Residential Autism Service	Focus Group	5 Females	Range 24–43 years Mean = 36.20 years, SD = 8.07	1 Senior Support Worker 4 Support Workers	Range 12–190 months Mean = 76.60 months, SD = 67.19
Community Mental Health Team	Focus Group	2 Females 1 Male	Range 38–53 years Mean = 46.67 years, SD = 7.77	1 Directorate Manager 1 Clinical Psychologist 1 Challenging Behaviour Nurse	Range 24–65 months Mean = 45.67 months, SD = 20.60
Autism Community Service	Focus Group	4 Female 1 Male	Range 24–45 years Mean = 29.60 years, SD = 8.73	5 Support Workers	Mean = 3 months, SD = 0.00
Learning Disability Residential	Focus Group	1 Female 4 Males	Range 24–52 years Mean = 34.80 years, SD = 13.24	1 Clinical Nurse Specialist 1 Staff Training Officer 1 Assistant Psychologist 2 Support Workers	Range 15–96 months Mean = 40.80 months, SD = 36.29
Community Autism Service	Focus Group	3 Females 2 Males	Range 21–53 years Mean = 37.40 years, SD = 14.36	3 Team Leaders 2 Support Workers	Range 11–60 months Mean = 43.00 months, SD = 18.64
Drug and Alcohol Rehabilitation	Focus Group	3 Females 2 Males	Range 28–61 years Mean = 44.00 years, SD = 14.05	1 Needle Exchange Assistant 1 Community Clinical Manager 1 Drug Rehabilitation Lead 1 Duty Worker 1 Clinical Lead	Range 1–112 months Mean = 33.20 months, SD = 46.26
Organic Inpatient and Older Adult Community Service	1:1 Semi-structured interview	1 Male	34 years	Registered Mental Health Nurse	54 months
Autism Community & Residential Service	1:1 Semi-structured interview	1 Male	53 years	Behaviour Nurse Specialist	120 months
Community Mental Health	1:1 Semi-structured interview	1 Female	40 years	Housing Support Worker	12 months

### Materials

An interview schedule was developed and utilised to guide discussion in each of the focus groups and 1:1 semi-structured interviews. The 6 guiding questions, as presented in Table 3, were asked within each interview and focus group. The only interview in which these questions were not asked was in an interview with participant 6 where the purpose was to discuss and verify the final iteration of the developed theoretical framework.

**Table 3:** Guiding questions used in the focus groups and interviews.

Interview Schedule
1) Could someone please provide an overview of the service here?
2) **How would you describe your work setting?**
3) What type of behaviours, displayed by care recipients, do you perceive to be challenging?
**4) What are the potential challenges that you experience within a normal working day?**
5) During a busy day at work, what coping strategies do you use to help you to get through the day?
**6) Is there anything that you think that health care organisations could do to help people who work in your profession?**

The topics derived from the participants’ responses to the 6 guiding questions then determined any additional questions that were asked in subsequent interviews and focus groups that were relevant to exploring the occupational demands of providing care for people who exhibit behaviours that challenge. This was to enable the iterative exploration of the commonalities and different perspectives of participants concerning the causes of and protective factors against work related stress when providing care for people who exhibit behaviours that challenge. A digital Dictaphone was also used to record the focus groups and interviews.

The Perceived Stress Scale (PSS) [27] was also administered prior to each focus group and interview to ascertain the levels of subjective stress experienced by participants. The PSS comprises of 10 items, where participants respond on a 5 point Likert scale ranging from 0 = never to 4 = very often. The scores derived from the PSS range from 0–40, with higher scores indicating greater levels of perceived stress experienced over the month prior to the date of data collection. Scores ranging from 0–13 are considered as low levels of stress, 16–26 moderate stress and 27–40 indicates high levels of perceived stress. The PSS taps into a single construct, perceived stress, with a Cronbach’s alpha being reported at 0.85.

### Researcher preparation

The first author (DR), had 3 years of experience of working as a support worker in Working Aged Adults Community Mental Health Services and an additional 3 years of experience as an assistant psychologist in Older Adult Community and Inpatient Services prior to data collection. Author DR also had previous experience of conducting qualitative research in healthcare settings prior to this study. This prior experience was useful throughout data collection and coding as Glaser and Strauss purport that researchers with relevant experience can have an understanding as to what data are relevant in informing the development of a given theory [24]. DR was responsible for conducting the 1:1 interviews/focus groups, transcribing the recordings and coding the transcripts. The first author had regular discussions with the research team (MW, MS and AM) throughout the collection/analysis of data and development of the theoretical framework as a means to negate the influence of any preconceptions towards the dataset. As a means to reduce researcher bias, the developing theory was also discussed as part of the interviews and focus groups with participants in the theoretical sample. The final iteration of the developed theoretical framework was discussed with participants 6, 46 and 47 to obtain confirmation that the developed theoretical framework provided valid explanations as to how work related stress can manifest or be reduced in health and social care professions.

### Ethics statement

This study was granted ethical approval from the Research and Ethics Committee at the University of Northumbria at Newcastle.

### Data collection and analysis

All participants were asked to attend a private room within their place of work to meet with the researcher. Participants were provided with an information sheet and a full briefing regarding the aims of the study. A group briefing was provided where focus groups were conducted, whereas for 1:1 interviews, participants were briefed on an individual basis by the researcher. Once the briefing had been completed, participants were asked to sign an informed consent sheet to document their agreement to take part in the study. Participants were then asked write down a behaviour that would be deemed most challenging when providing care and then complete the Perceived Stress Scale [27]. The researcher notified participants that the digital Dictaphone would be switched on to then begin the recording of the focus group or 1:1 semi-structured interview. A semi- structured interview schedule was used, comprising of open- ended questions, as a means to direct the discussion of the focus groups/1:1 interviews that was relevant to the aims of the study. Socratic questioning was also used to avoid participants from using single word answers, to encourage elaboration on any discussion points that had been made and to ensure that responses were relevant to the research aims. The focus groups and 1:1 interviews lasted approximately 90 minutes each. Once the focus groups/1:1 interviews had ceased, participants were notified the Dictaphone had been stopped/switched off, were then provided with a debrief sheet and thanked for their time.

The process of comparative analysis was adhered to, in accordance with CGT methodology [24], as a means to explore the experiences of participants who worked across a number of different healthcare and social care settings. The initial purposive sampling phase consisted of coding the transcripts derived from the focus group and 1:1 semi-structured interviews to ascertain the common and conflicting perspectives of participants, across the various settings, concerning how work related factors potentially impacted stress levels experienced. This stage consisted of collecting sufficient data to identify/saturate categories that were relevant in explaining the conditions under which stress can manifest or be reduced in health/social care professions. The purposive sampling stage also consisted of saturating categories and identifying professional groups who had experience of managing behaviours that challenge in health and social care settings, who were to be recruited in the theoretical sample. Theoretical sampling consisted of conducting further focus groups/interviews with suitable health and social care professionals as a means to refine the developed categories, core category and to formulate the overall theoretical framework. The beginning of each focus group or 1:1 semi-structured interview, within the theoretical sampling phase, consisted of showing and explaining the developed theoretical framework to participants before commencing with the interview schedule (Table 3). This was to verify, with participants, as to whether the developing categories/core category in the theoretical framework was providing valid representations of how work related stress could both manifest and also be reduced in health/social care professionals when caring for people who exhibit behaviours that challenge. The final 3 interviews of the theoretical sampling provided opportunities for participants to discuss and verify that the final iteration of the theoretical framework was conducive to providing a valid explanation as to how stress can occur or be reduced when providing care for people who exhibit behaviours that challenge.

### Descriptive statistics

*Behaviours deemed to elicit the most challenging situations for frontline staff*. Table 4 provides a summary of the types of distressed behaviours that participants deemed as being most challenging when providing direct health and social care interventions.

**Table 4:** A breakdown of the distressed behaviours that participants reported as being most challenging when exhibited by care recipients.

Behaviour deemed to elicit the most challenging situations in the workplace	n	%
Verbal Aggression	18	38.3
Physical Aggression	11	23.4
Self-Injurious Behaviours	7	14.9
Apathy to engage with treatment interventions	5	10.6
Repetitive Vocalisations	3	6.4
Lying (being deceitful)	1	2.1
Vandalism	1	2.1
Spitting	1	2.1

Table 5 also provides descriptive statistics to illustrate the perceived stress levels experienced by participants at the time of data collection. In accordance to the cut-off values for the Perceived Stress Scale [27], the observed mean value of 17.20 is indicative of moderate levels of perceived stress.

**Table 5:** Descriptive statistics of responses provided by participants, across both the purposeful and theoretical sample, on the Perceived Stress Scale [27].

Participant Group	n	M	SD
**Perceived Stress Scale**	47	17.20	6.32

The Perceived Stress Scale [27] has been used previously as a marker to illustrate self-reported stress levels in people who provide informal care for people who exhibit behaviours that challenge. Gallagher and Whiteley [28] published perceived stress scores for parent carers of children with intellectual disabilities who exhibit distressed behaviours, such as verbal aggression and social withdrawal, with n = 70, M = 10.20, SD = 2.73.

## Results

**Fig 1:**
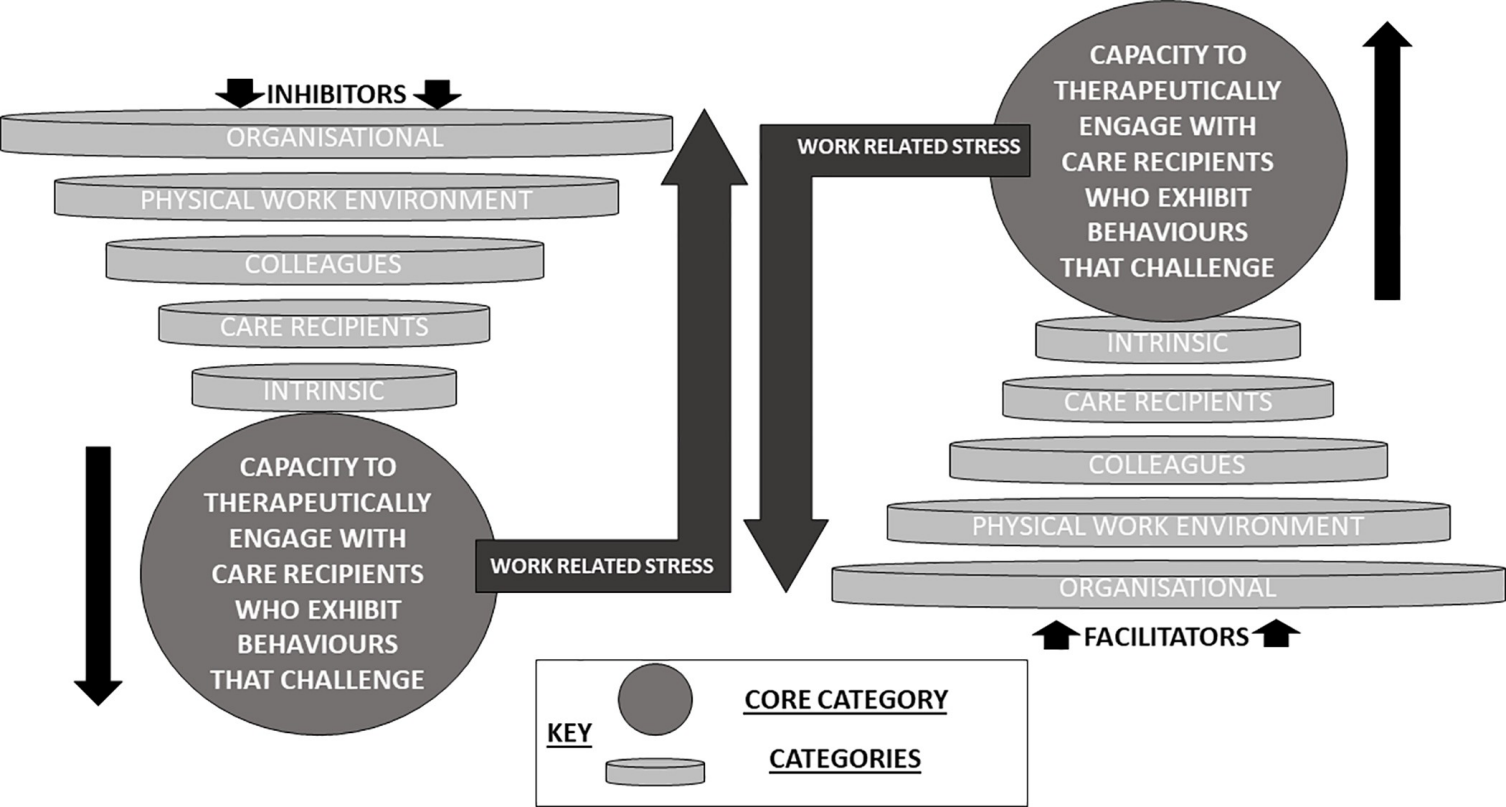
Illustration of Therapeutic Engagement Stress Theory (TEST).

A central issue that explained the levels of work related stress experienced by health and social care professionals was their capacity to engage therapeutically with care recipients who exhibit behaviours that challenge, which could be influenced by 5 categories. Those categories were 1) organisational, 2) physical work environment, 3) colleagues, 4) care recipients and 5) intrinsic factors. Within each category, participants articulated that there were factors that reduced their capacity to engage therapeutically with care recipients who exhibited behaviours that challenge, which could increase levels of work related stress. However, participants stated that there were also potential strategies within each of the 5 categories that could support frontline staff in their delivery of caring interventions for care recipients, which could then reduce work related stress. Fig 1 illustrates the process of how an interplay between categories could influence the extent to which frontline staff can engage with care recipients who exhibit behaviours that challenge, which can then determine levels of work related stress experienced.

**Fig 2:**
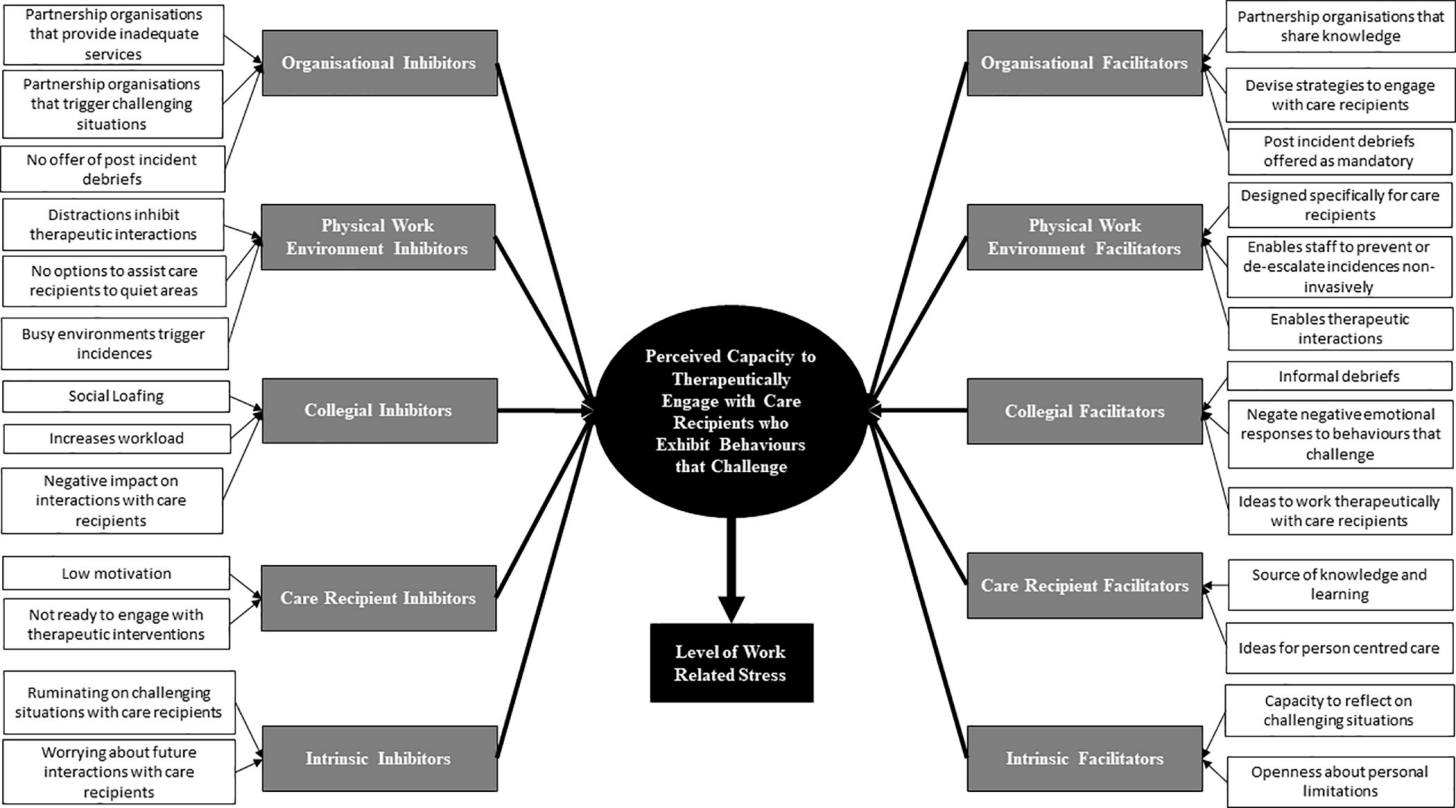
Coding tree for Therapeutic Engagement Stress Theory (TEST).

Fig 2 also provides an illustration of the coding tree to demonstrate how the codes informed the development of categories, core category and overall theoretical framework.

### 1. Organisational

Participants discussed how the overarching cultures, policies and procedures of employing organisations could affect their ability to provide care for people who exhibit behaviours that challenge.

#### Organisational factors that inhibit the capacity to engage with care recipients

Participants acknowledged that it was a requirement to collaborate with external organisations in order to meet the holistic health and social care needs of care recipients. However, some participants suggested that working with partnership organisations that did not communicate patient specific knowledge or assume their responsibilities to deliver health/social care services, could inhibit frontline staff from successfully engaging with care recipients.

‘***Participant 48*:** *When you go into working partnerships*, *that’s a massive stressor as well because you go into partnership with a service that say that they can provide X*, *Y and Z and then when you’ve signed the dotted line*, *they can’t provide X*,*Y and Z*, *so your service still feels it*. *The patients…they don’t understand that*, *they don’t know what’s going on in the background*, *they don’t get that the other organisation can’t do what they said they would do so then that impacts the patients*. *They don’t make that connection so then people get angry with us*. *We don’t explain to them that we’ve lost 30% of our workforce in the last year by putting more and more money into other organisations that said they could do what they were going to do*. *So working in partnership is a massive stressor for our services*.’

**(Participant 48 –Drug Rehabilitation Lead)**

The extract above suggested that partnership organisations that do not assume anticipated caregiver responsibilities could trigger care recipients to exhibit overtly aggressive behaviours towards frontline staff. Thus, work related stress can be elicited when frontline staff are inhibited in their ability to deliver care effectively when care recipients exhibit aggressive behaviours due to partnership organisations failing to deliver their services effectively.

**Effective communication with partnership organisations.** It was suggested that working in a network of services that provided outlets to share caregiver responsibilities and patient specific knowledge could inform strategies to engage therapeutically with care recipients known to have exhibited behaviours that challenge, which was helpful in offsetting stress.

‘***Participant 47*:** *What we’ve found is that you really need a thorough introduction from the current people [external organisation] working with that individual*. *A good relationship with the person making the referral*, *usually a Social Worker*, *means that we can actually present to staff what could potentially happen if you don’t follow the plan*. *People who have been referred here have had great difficulties and have had chequered lives*, *they have gone through all sorts of trauma and they come to us and is it any surprise that they present with the range of behaviours that they do*? *But we’ve had that liaison [with the external organisation] and we’ve had the time with the person making the referral*. *Ultimately it’s protecting the staff which is then giving the best service to the new person coming in*.*’*

**(Participant 47—Behaviour Nurse Specialist)**

Participants indicated that working alongside partnership organisations that fulfilled their collaborative duties and had good communication links, could inform the development of care plans and strategies to engage with care recipients who have previously exhibited behaviours that challenge. Such care planning could avoid triggering incidences of behaviours that challenge, which would help frontline staff in their capacity to deliver caring interventions and thus negate the onset of work related stress. This would suggest that effective communication and collaboration with partnership organisations could offset work related stress through informing frontline staff on strategies to engage with care recipients.

**The mandatory offer of post-incident debriefs.** Participants also suggested that working for organisations that offer employee frontline staff post incident debriefs, as mandatory, can be helpful in facilitating reflective practices to ascertain strategies to prevent further incidences of behaviours that challenge.

“***Interviewer*:** *Do you find that helpful*, *having the debriefs*?

***Participant 12:*** *Yes, I think it is. You can look at what might have worked better or worked well. If it worked well, then you can use that approach in the future to stop further incidences. Or if something didn't work, you know not to do that again*.

***Interviewer:*** *So it is good having debriefs to learn and try and prevent further incidences of behaviours that challenge?*

***Participant 12:*** *Yeah, it helps to reflect back on it I suppose. Our debriefs are normally meeting with our ward manager. He would ask “What happened?” and we would just describe the incident and we would go from there. He would ask, “So why did you do that? Do you think that worked well?’*

***Interviewer:*** *Do you find that having that debrief is good for emotional support too?*

***Participant 12:*** *Yeah, I think that it does. You feel that sometimes after an incident…you are really hot and you are really like “oh my god” and you have that sigh of relief that it’s come to an end. I think just having 10 or 15 minutes away off the floor, as it were, is good. It gives you a chance to calm because if an incident ends up in a restraint or seclusion, the adrenaline starts pumping and it is good to just have that bit of time away and reflect on what happened”*.

**(Participant 12: Staff Nurse Mental Health Inpatient Service)**

Participants suggested that the process of having a formal debrief with a senior member of staff immediately following an incident of behaviour that challenges can be helpful in facilitating reflective practice, achieving homeostasis and continuing therapeutic work with care recipients concerned. However, some participants discussed the impact on employee wellbeing when organisations do not have strict protocol to ensure that staff receive post incident debriefs immediately following incidences of behaviours that challenge.

“***Participant 52*:** *I would say that [debriefs] is something you can’t always do because of the demands in terms of time*. *An example of that was dealing with something the other week*. *I had somebody kicking off in reception and very violent in terms of the way that they were talking*, *behaving and being threatening*. *It was really difficult and I went home that day feeling pretty awful*. *I actually shut down that weekend at home because it was just one too many incidences*. *Normally with those things I would be fine*. *But for some reason*, *that one got to me and I’m not really sure why*. *But I went home*. *I felt wobbly walking up the street and I was worried that I might bump into them outside in the street*. *I rang home and spoke to my bloke*, *not to tell him about what happened as such*, *but just to talk to somebody while I walked to the bus stop*, *until I got onto the bus to go home*, *until I felt safe”*.

**(Participant 43 –Duty Worker in Drug and Alcohol Rehabilitation Service)**

The above extract would suggest that the implications of not offering post incident debriefs as mandatory has the potential to cause staff to worry about or avoid further interactions with care recipients who have exhibited behaviours that challenge and become withdrawn, which could elicit work related stress.

### 2. Physical work environment

Participants suggested that employing organisations had an obligation to provide staff with appropriate physical working environments and resources that were conducive to preventing incidences of behaviours that challenge as a means to negate the onset of work related stress.

#### Physical work environments that inhibit therapeutic interactions with care recipients

Some participants articulated how work related stress can manifest when their capacity to therapeutically engage with care recipients was inhibited by their physical working environment.

“***Interviewer***: *Do you think you can do that [prevent or distract care recipients from exhibiting behaviours that challenge] on the ward where you are working now*?

***Participant 46***: *Not really, it depends on the ward because some are better than others. You get wards that were built for other purposes. So you’ve got one massive long corridor with bedrooms coming off it and one big lounge*.

***Interviewer:***..*and that’s not appropriate?*

***Participant 46***: *It’s not designed specifically, yeah*.

***Interviewer***: *Why do you think that environment is potentially stressful for staff?*

***Participant 46***: *Because there’s not much to play with. There’s not much for members of staff to be able to become creative with it and look how they can facilitate different experiences and activities for the patients. You need a good room to get away and get some good work done with the patients. But that doesn’t exist*.

**(Participant 46—Registered Mental Health Nurse)**

The extract above indicated that working within a physical environment, which was not designed specifically for a particular patient group, may inhibit staff from being able to employ effective strategies as a means to prevent or de-escalate incidences of behaviours that challenge in a non-invasive manner. The process of providing care in long-term care institutions that are devoid of providing health/social care professionals with opportunities to use person centred approaches, could potentially inhibit staff from implementing interventions that are convergent with the specific needs of care recipients. Furthermore, work related stress may occur for frontline staff who are unable to modify care recipients’ physical environments accordingly as a means to prevent incidences of behaviours that challenge from occurring.

#### Safe prevention and de-escalation of behaviours that challenge

Participants articulated that providing care within physical work environments that are specifically designed to facilitate the assessment and treatment of particular patient populations could be beneficial in assisting staff to implement preventative strategies to negate incidences of behaviours that challenge and engage therapeutically with care recipients.

“***Participant 21*:** *What helps the stress is working in the building that's been specifically designed for the client group*. *We have soundproofing in the clients’ rooms*. *Clients have separate flats and we have lots of communal areas*. *The building I work in is specifically designed for our client group which takes away some of the stress because when you are talking about the incidences that are happening in communal areas*, *it is designed that they already live within their flats*. *They are free to move about and we assist them in that way [living independently]*. *Working in a building*, *that is so highly designed*, *lessens all that stress*. *Where I worked previously*, *things were 20 times worse for myself and I was stressed because of that*. *Where I work now has been designed with a purpose and it shows because all of that thought process has gone into it before anybody even moved into it*. *It has had a massive impact on how we deal with incidents”*

(Participant 21—Support Worker in an Autism Residential Service)

“***Participant 46***: *If you’ve got the environment to work with*, *then you can prevent incidences from occurring*. *You can use all of those proactive approaches that’s identified through the person centred care planning*. *If you’ve got the right environment*, *then you have got a really*, *really good resource to be able to effectively communicate [with service users]*, *do your job and offer care*. *You can just adapt to situations better*. *Reassure*, *distract*, *orientate*, *you can use all the different tools to prevent incidences from happening*. *If you can change the environment to a way that it needs to be for the person with dementia*, *then you are laughing really*.*”*

**(Participant 46—Registered Mental Health Nurse)**

It appeared that work related stress could be negated when working within settings that have been designed to accommodate care recipients to engage in meaningful activities, which was also beneficial in assisting frontline staff to implement person centred approaches to prevent incidences of behaviours that challenge. Thus, the process of using person centred approaches to prevent or de-escalate behaviours that challenge was also purported to assist frontline staff to engage therapeutically with care recipients, which was beneficial in reducing the onset of work related stress. This would suggest that working in physical environments that facilitate therapeutic engagement with care recipients who exhibit behaviours that challenge, through provision of the necessary resources that meet patients’ needs, could serve to buffer work related stress for health/social care professionals.

### 3. Colleagues

The category of ‘colleagues’ encapsulated participants’ experiences of how their peers could influence work related stress levels through either facilitating or inhibiting the delivery of therapeutic interventions for people who exhibit behaviours that challenge.

#### Colleagues who avoid caregiver duties

Some participants suggested that working alongside colleagues who do not contribute to caregiver duties could increase the workload for other members of frontline staff, which could inhibit therapeutic interactions with care recipients and contribute to the manifestation of work related stress.

‘***Participant 46*:** *It breeds contempt when you don’t feel supported by your team*. *Subconsciously or somewhere down the line*, *it can have a negative impact on my work with patients because if you are running around doing all of the work*, *you are going to be stressed*. *Therefore*, *you are not going to get that effective interaction with the patients or be able to spend as much time with them as you would do to offer them an intervention*. *You might be a bit more rushed*. *Then you’re a little bit hacked off or irritable*. *You’re not being able to have that effective communication because your tone of voice or facial expression with that patient isn’t what it would be if you actually felt supported with the work load’*.

**(Participant 46 –Registered Mental Health Nurse)**

Colleagues who do not effectively contribute in the workplace can elicit perceptions of injustice and increase burden in other areas of the workforce, which can negatively affect the way in which the more motivated staff members interact with care recipients. Thus, work related stress can be triggered in frontline members of staff who perceived that their capacity to engage with care recipients is inhibited by working alongside unsupportive colleagues.

#### Supportive and informal debriefs with colleagues

Some participants stated that working alongside colleagues who are supportive and provide informal debriefs following incidences of behaviours that challenge can be effective in ensuring the continued delivery of interventions for care recipients concerned and reducing stress.

‘***Participant 18*:** *We had a service user who was threatening to jump out of his bedroom window and it was awful*. *It was an absolutely horrendous shift*. *But I was on with two very experienced members of staff*. *I felt I was absolutely useless and my stress levels were off the charts and I said “I can't deal with this*, *I can't do this job” after that incident*. *But sitting down with those two members of staff and talking everything through helped me to understand that service user because he was new to everyone as well*. *But I did have thoughts of not going back into work and having to face that one service user again*. *I was quite new but they just gave me reassurance that I hadn't done anything wrong*, *I handled it really well*. *I was so stressed when I entered the building the next day*, *but after half an hour*, *I actually worked with that same service user and we had a brilliant night*. *So it does help when you have those people [colleagues] around you’*.

**(Participant 18—Support Worker in an Autism Residential Service)**

The above extract provides an illustration as to how behaviours that challenge, as exhibited by care recipients, can cause stressful situations in the workplace for health and social care professionals. Behaviours that challenge also have the potential to fracture a therapeutic relationship between the carer and the care recipient. However, discussion with supportive colleagues can potentially assist frontline staff to develop and implement strategies to maintain/improve their caregiver practice, which is a process that can reduce work related stress. As a strategy to reduce work related stress, it is necessary that health/social care organisations harness cultures that elicit collaboration between members of staff to enable the successful delivery of caring interventions for people who exhibit behaviours that challenge.

However, it was also suggested that working with colleagues who do not show support towards frontline staff who have been involved in incidences of behaviours that challenge can also potentially elicit or perpetuate work related stress within health and social care professionals.

“***Participant 23*:** *I could be sent in on a morning to get her [a care recipient] up and do her personal care and then again on a night-time where she might have wanted somebody else to go in and do it on a night-time*. *She didn’t want to see me again*. *She would just lash out and hit and it was always me who got it because I was stuck with her day after day*. *I think that at the time*, *we had certain staff members who thought it was funny*. *My hair was just was coming out in clumps*, *she had hold of me that much*. *Some certain members of staff just laughed and thought it was funny*. *I did actually open my mouth and say “Right that's it*, *enough is enough*. *I am not doing it anymore*. *I am not working with that morning*, *noon and night”*.

**(Participant 23—Support Worker in Residential Autism Service)**

The current study would also indicate that colleagues who undermine the difficulties that some frontline staff experience when encountering incidences of behaviours that challenge can be perceived as workplace bullying and may elicit work related stress to the extent of requiring to take sick leave. Thus, working alongside colleagues who do not empathise with the difficulties of encountering incidences of behaviours that challenge could disrupt the capacity for health and social care professionals to engage in their occupation.

### 4. Care recipients

Participants’ articulated how direct interactions with care recipients who exhibit behaviours that challenge could affect their capacity to successfully deliver therapeutic interventions and thus influence levels of work related stress.

#### Care recipients who are not motivated to engage with therapeutic interventions

Some participants articulated how work related stress can manifest when providing caregiver duties for care recipients who do not engage with therapeutic interventions.

‘***Participant 8***: *You can get a lack of motivation [from care recipients] that can be due to medication or their illness*. *I was working with a service user to help them work out their finances*. *I made a lot of effort and it all came to nothing and that can be challenging*.

**Participant 4**: If you are supporting somebody longer term, just working on behaviour change, you can get trapped into quite negative cycles. You are giving people [care recipients] strategies that they can use and then they don't use them. You're giving people things to do and strategies to use and then you meet with them the next week and you are asking “Have you done what we set last week?” and then they say “No I have not”…

**Participant 7**:…and they [care recipient] say “I have tried it for two weeks and it hasn't worked”.

**Participant 4**: Keeping people [service users] constantly motivated is quite challenging’.

**(Participant 4 –Senior Support Worker within a Community Mental Health Service**

**Participants 7 & 8—Support Workers within a Community Mental Health Service)**

This suggests that health and social care professionals can experience work related stress when perceiving that their capacity to deliver therapeutic interventions is inhibited by care recipients who appear unmotivated or ambivalent to engaging with treatment. As stated, collaboration with partnership organisations, working in physical environments that enable safe prevention/de-escalation of challenging situations and supportive colleagues can serve to buffer stress through increasing the capacity for frontline staff to engage therapeutically with care recipients. As a means to reduce work related stress, it would therefore be necessary for health and social care professionals to tap into factors that serve to facilitate the delivery of therapeutic interventions when encountering care recipients who present as unmotivated or ambivalent to engage with services.

#### Learning how to successfully deliver interventions through direct interactions with care recipients

However care recipients, who were open to engage with staff members, were viewed as being beneficial in acquiring work related knowledge and informing strategies to deliver therapeutic interventions in a way that met the bespoke needs of each care recipient.

“***Participant 4***: *I sometimes think back to when I first came to work here and I learnt a lot about the clinical side of mental health and diagnoses*. *Actually*, *when I think about it now*, *it just makes me shudder because the people who I have learnt the most from and learnt the most useful information is from the actual people [care recipients] who come here*. *Just being sat down and listening to people and listening to their stories and actually figuring out what to do based on what people say*. *Some people do need very specific support and very specific care and it helps to really understand that person's background and some of the things that they are struggling with”*.

**(Participant 4 –Senior Support Worker within a Community Mental Health Service)**

### 5. Intrinsic qualities of health and social care professionals

Participants suggested that the intrinsic qualities of frontline health/social care professionals could either inhibit or facilitate their delivery of therapeutic interventions to care recipients who exhibit behaviours that challenge and influence levels of work related stress.

#### Repetitive negative thought processes

Work related stress can occur for health/social care professionals who are prone to experiencing repetitive negative thoughts concerning previous and potential future interactions with care recipients who exhibit behaviours that challenge.

‘***Participant 4***: *I have had plans and I have cancelled them because I just thought I will be an absolute misery*, *which isn’t great because you retreat into your cave for a bit*. *Sometimes it's all very well to say “Oh well just stop thinking about those things”*, *but it is really hard*. *It is really hard when you care about the people [care recipients] that you work with*. *It is really hard to switch off from that sometimes*. *It is horrendous*. *I go home and dwell on it*. *You question your input and you say “I should have helped that person more*, *I should have done that differently”*. *You go over it and it can make you feel quite helpless’*.

(Participant 4 –Senior Support Worker within a Community Mental Health Service)

‘***Participant 20***: *Say you went out to [assist a care recipient to attend] the cinema*, *then it can be a lot more stressful*. *Even before you have set off*. *You are taking somebody that you know that can display a challenging behaviour*. *You know that you are taking them out into this public situation and obviously you are worried for them*, *you are worried for yourself as well*. *You are worried about what Joe Public are going to do’*.

**(Participant 20—Support Workers in Residential Autism Service)**

The quotes above would indicate that health/social care professionals who have a tendency to engage in repetitive negative thinking, through ruminating on past incidences or worrying about future interactions with care recipients who have exhibited behaviours that challenge, could be prone to work related stress.

#### Having self-awareness and disclosing personal limitations

Having insight to and disclosing personal limitations appeared to be an intrinsic factor that could help with identifying areas to improve as a practitioner in order to engage with people who exhibit behaviours that challenge and thus reduce work related stress.

‘***Participant 25***: *It’s having that self-awareness of what you need to manage in those situations [of behaviours that challenge] and support you*. *You do need to ask for that support and go for and be willing to be open about it*. *Not everyone has that self-awareness of being honest about what they need to develop or what they are comfortable with*. *I think it’s a big thing to say “No*, *in actual fact I am not good at that and I need to do something about it”*. *I think you have to be very open about yourself in that way’*.

**(Participant 25 –Challenging Behaviour Nurse)**

The ability to disclose limitations can provide opportunities to engage in reflective practices and openly discuss strategies with supportive colleagues in order to successfully engage with care recipients who exhibit behaviours that challenge and thus reduce work related stress. It could be that organisational procedures that encourage working practices, such as informal debriefs with colleagues, may help to elicit constructive reflection on incidences of behaviours that challenge, negate repetitive negative thoughts, and thus offset work related stress.

## Discussion

The theoretical framework derived from this study, entitled Therapeutic Engagement Stress Theory (TEST), illustrates how the perceived capacity to therapeutically engage with care recipients who exhibit behaviours that challenge is a central issue that can determine the levels of work related stress experienced by health and social care professionals. The results also demonstrate how the integration of categories; 1) organisational, 2) physical work environments, 3) colleagues, 4) care recipients and 5) intrinsic thought processes, can influence health/social care professionals in their perceived capacity to engage therapeutically with care recipients.

Participants articulated how the process of partnership working with external health/social care organisations, can impact work related stress through influencing their capacity to engage with care recipients. There have been a number of health initiatives in the UK that have advocated for the collaborative working between health and social care services, one example being the NHS Five Year Forward View [29], to ensure the provision of holistic care packages for people who have multiple care needs. It has been advocated that the contribution of expertise from multiple services is essential in ensuring the delivery of holistic care packages that successfully meet the biological, psychological and social needs of services users [30]. Collaboration with partnership organisations that consists of sharing resources, [31], caregiver responsibilities [32] and communicating information that may assist with successful delivery of care [33] may facilitate staff in providing care for people who exhibit behaviours that challenge, thus offsetting work related stress.

The results also suggested that the way in which organisations implement support, following challenging situations with care recipients, can influence the stress levels experienced by frontline staff. The provision of debriefs for staff immediately after they have encountered incidences of behaviours that challenge may be conducive in ensuring employee safety and wellbeing [34]. However, previous research has also shown that not all healthcare organisations, that provide assessment and treatment for people who exhibit behaviours that challenge, ensure the delivery of post incident debriefs for frontline staff as mandatory [35]. It has been suggested that post incident debriefs that consist of root cause analysis can be beneficial in facilitating frontline staff to engage in problem solving as a means to identify and consider strategies to negate triggers for behaviours that challenge within mental health inpatient services [36]. Thus, failure to provide post-incident debriefing could be missed opportunities for organisations to support staff to engage in reflective practices/problem solving as a means to address the behavioural symptoms of service users and offset unhelpful rumination on incidences of behaviours that challenge. However, it must be acknowledged that the way in which debriefs are offered and conducted requires thorough consideration given that discussion of traumatic events, such as incidences of behaviours that challenge, can potentially elicit further trauma [37] within staff involved in challenging incidences. In addition, some healthcare organisations may not use standardised guidelines when providing staff with post incident debriefs [35]. There is also evidence to suggest that post incident debriefs should not be used routinely in order to avoid re-traumatisation, and that ongoing emotional and instrumental support should be provided as an alternative to ensure the wellbeing of frontline staff who have been involved in challenging incidences within the workplace [38] Thus, there is a need to develop a clear understanding as to how organisations can support frontline staff immediately following incidences of behaviours that challenge in order to ensure emotional wellbeing, offset work related stress and facilitate employees to engage in reflective practices on their therapeutic work.

Physical work environments were also cited as being influential in how health and social care professionals were able to employ person centred strategies to engage with care recipients and safely prevent incidences of behaviours that challenge. Person centred care is a philosophy of health and social care which advocates that therapeutic interventions should be informed by the values and tailored to meet the idiosyncratic needs of each individual care recipient [39]. In relation to dementia care, Kitwood [40] developed a model of person centred care, which stipulated that healthcare environments should provide opportunities to engage in activities that are meaningful and converge with the personal interests of care recipients. Within nursing homes for people with dementia, the ability to distract care recipients from encountering triggers, through engaging in meaningful activities, can be effective in reducing symptoms of agitation and preventing incidences of behaviours that challenge [41]. Ensuring that care recipients have access to private areas when taking residence within a long-term healthcare setting can also be conducive in negating agitation and aggressive behaviours [42]. Physical workplace environments that can be modified to provide opportunities for privacy and facilitate activities that are relevant to the personal values of care recipients could assist frontline staff to implement person centred care strategies to prevent incidences of behaviours that challenge. Thus, health/social care settings that comprise of features that provide staff with outlets to engage therapeutically with care recipients may also be conducive in offsetting work related stress within formal carers.

Levels of collegiate support was also discussed as having an influence on the way in which frontline staff were able to provide care for people who exhibit behaviours that challenge. Working alongside colleagues who avoid caregiver duties, which was identified as inhibiting the successful delivery of care, can be explained by the phenomenon of Social Loafing, which suggests that individual members of staff reduce their effort when working as part of a team as opposed to when working independently on a given task [43]. It has been recognised that the colleagues who exhibit the tendencies associated with social loafing can cause members of staff, who are motivated to achieve organisational tasks, to increase their work related effort in order to counteract the more apathetic team members [44]. However, participants stated that working alongside colleagues who were supportive and collaborative could facilitate the successful delivery of caring interventions. Social support has been defined as the networks that provide psychological and material resources that facilitate the successful management, offsetting or overcoming of particular stressful situations [45]. Previous research has suggested that the process of discussing work related issues with colleagues, who are experiencing similar occupational demands, can be conducive in offsetting stress and burnout within healthcare professions [46]. Thus, informal debriefs and collaboration with colleagues may provide the necessary support that is conducive in negating the work related stress that can coincide when encountering care recipients who exhibit behaviours that challenge [47]. This would suggest that informal debriefs with colleagues may provide sources of social support that enable health/social care professionals in their continued delivery of care to people who have previously exhibited unhelpful behavioural symptoms and thus offset work related stress.

It was also posited that the levels at which care recipients engage with health and social care services, could influence the perceptions of frontline staff in their capacity to deliver therapeutic interventions. It was stated that care recipients can be a source of knowledge that can facilitate frontline staff in delivering interventions that are person centred. In the field of Psychotherapy, Carl Rogers [48] posited that care recipients have the greatest insight to their experience and should inform the way in which therapists implement therapeutic interventions. The current study also illustrated the importance of listening to and identifying the needs of care recipients in order to enable frontline health/social care professionals to deliver therapeutic interventions in an effective manner. Training interventions, that have been effective in improving efficacy and knowledge in the safe prevention/de-escalation of behaviours that challenge, have shown to elicit acute beneficial effects in negating burnout within professional carers of people with dementia [49]. This would suggest that access to information, increasing work related knowledge, and improving efficacy in the prevention of behaviours that challenge, could all serve as protective factors against work related stress. Therefore, care recipients who are open to disclosing their needs and are willing to engage with health/social care services, could facilitate frontline staff in their capacity to engage therapeutically, achieve their caregiver duties and thus negate work related stress.

The propensity to have repetitive negative thoughts was also discussed as having detrimental consequences on health/social care professionals in their ability to engage with care recipients who exhibit behaviours that challenge, which can elicit work related stress. Rumination is a term used to describe repetitive negative thought processes on incidences that have occurred in the past [50]. Worry has been defined as the repetitive thinking and fixation on the potential for future negative events to occur [51]. Participants indicated that health/social care professionals who have a tendency to engage in repetitive negative thinking, through ruminating on past incidences or worrying about future interactions with care recipients who have exhibited behaviours that challenge, could be prone to work related stress. However, reflective practice was illustrated as a thinking style that could facilitate frontline staff in their ability to devise strategies to successfully engage with care recipients who exhibit behaviours that challenge. The process of reflection can consist of appraising personal input on a given task, identifying areas for improvement and implementing the required changes in order to enhance professional practice [52]. Within healthcare professions, it has been purported that the ability to reflect on personal practices can be fundamental to the process of learning, acquiring knowledge and becoming a more effective practitioner [53]. It would be of interest to ascertain if interventions that encourage reflective practices were conducive to reducing negative thought processes, increasing capacity to engage with care recipients who exhibit behaviours that challenge and negate work related stress.

It must be acknowledged that all of the participants who took part in the study were employed and engaged in a health/social care professional profession at the time of data collection. In 2017, 38% of 468,712 NHS staff reported to have experienced ill health due to work related stress [54]. This may explain why the sample in the current study, on average, was found to have moderate stress levels when measured using the Perceived Stress Scale. However, the Perceived Stress Scale is a general measure for subjective stress [27] and does not specifically measure work related stress. Therefore, it cannot be determined as to whether the moderate stress levels observed to summarise the sample in the current study, were due to the specific occupational demand of caring for people who exhibit behaviours that challenge. It could be argued that the current study should have sought the articulated experiences of health/social care professionals who were not engaged in their profession, due to work related stress, at the time of data collection. This may have been beneficial in ascertaining ‘in the moment’ perspectives as to what aspects of the occupation cause work related stress to the extent of requiring to take sick leave from work. However, there would be ethical issues recruiting participants who were on a leave of absence due to work related stress, given that reflecting on traumatic incidences without having the opportunity to reappraise the events in a helpful manner, can potentially elicit re-traumatisation and have negative consequences on wellbeing (Littrell, 2009). However, all of the participants who took part in the study stipulated that they had first-hand experience of providing health or social care to people who exhibit behaviours that challenge. Existing literature [2, 3, 5]clearly suggests that the occupational task of encountering incidences of behaviours that challenge in health and social care settings can be stressful. Thus, it is reasonable to suggest that the participants who took part in this study had relevant experiences in order to give valid insights to the causes of and protective factors against work related stress when caring for people who exhibit behaviours that challenge.

A limitation of the current study is that some work related factors, which may influence the capacity for staff to engage therapeutically with care recipients who exhibit behaviours that challenge, may not have been revealed or fully explored in the current study due to the methods of data collection. It has been posited that the social status of and the pre-existing relationships between individual participants can impact the issues that are discussed and disclosed within focus group settings [55]. Some of the focus groups in the current study consisted of having senior members of staff being present, thus more junior employees may have only articulated socially desirable information and could have been inhibited to disclose genuine issues relevant to occupational stress. Therefore, it could be that some work related factors, which may impact the capacity for staff to successfully engage with care recipients, were not articulated by some participants in the current study due to the inhibition to disclose such information in a focus group setting with work colleagues.

It must therefore be acknowledged that the interplay between the 5 illustrated categories (Figs 1 and 2) and how they influence work related stress through impacting the capacity of engaging therapeutically with care recipients who exhibit behaviours that challenge, can be bespoke to each individual health/social care professional. For instance, some health and social care professionals may be inhibited in their capacity to deliver therapeutic interventions due to unhelpfully ruminating on previous interactions with care recipients who exhibit behaviours that challenge; a process that could manifest into work related stress. However, such issues could be resolved through accessing the support of colleagues through informal debriefing and encouraging reflective practices to devise strategies to increase capacity to engage therapeutically with care recipients who have exhibited behaviours that challenge, and thus reduce stress. As a means to offset work related stress, it is therefore necessary utilise TEST as a means to ascertain the bespoke factors that would facilitate health/social care professionals to successfully engage care recipients in a therapeutic manner. In their seminal paper concerning Grounded Theory methodology, Glaser and Strauss [23] stated that categories and core category should be clearly demarcated to the extent that they can be operationalised using quantitative measures. TEST provides a framework that could be investigated, using quantitative methods, to ascertain if the core category (capacity to therapeutically engage with care recipients exhibiting behaviours that challenge) mediates any relationships between the 5 stipulated categories and work related stress. It is suggested that using screening tools that consist of items that are specific to a particular occupation can be more beneficial in identifying effective coping strategies to negate stressors, than when administering generic stress measures [56]. The next step of this research programme will therefore consist of developing quantitative measures, using Factor Analysis techniques, as a means to operationalise the categories and core category in TEST. Thus, TEST will be used to develop a stress screening tool that could be used in health/social care services to ascertain how organisational, the physical work environment, colleagues, care recipients and intrinsic thought processes either facilitate or inhibit frontline staff in their capacity to engage therapeutically with patients.

A screening tool, as underpinned by TEST, could be used by Occupational Health Services (OHS), within healthcare organisations such as the National Health Service, in order to identify bespoke methods to support frontline staff in their successful delivery of care [4]. Supervisors, or managers, have also been recognised as having a key role in identifying the occupational health needs of frontline staff and ensuring the appropriate support mechanisms are in place to ensure the wellbeing of employees [57]. Occupational support that addresses the idiosyncratic needs of frontline healthcare staff who present as or disclose to be experiencing work related stress, have shown to be most effective in reducing stressors within caring professions [58]. Thus, the TEST model could be applied by professionals, such as OHS staff, to identify bespoke features within 1) organisational factors, 2) work place settings, 3) colleagues, 4) care recipients and 5) intrinsic thought processes that could be used as bespoke strategies to facilitate staff in their capacity to deliver therapeutic interventions and thus reduce stress.

The current study aimed to develop a theoretical framework that illustrated a common issue that could explain how stress manifests in professions that consist of providing health and social care services for people who exhibit behaviours that challenge. The results led to the development of TEST which posits that the perceived capacity to engage with care recipients who exhibit behaviours that challenge is a key factor that can determine the levels of stress experienced by frontline staff who care for people who have behavioural symptoms. Future research will consist of developing quantitative measures that ascertain how organisational factors, work place settings, colleagues, care recipients and intrinsic thought processes specifically influences the capacity for frontline staff to successfully engage with care recipients. This future research will aim to develop a stress screening tool that could be applied to inform implementation of bespoke strategies that focus on supporting staff to engage therapeutically with care recipients who exhibit behaviours that challenge as a means to reduce stress in health and social care professions.

## Supporting information

S1 File (DOCX)

S2 File (DOCX)
