# Supplementary material for: A grounded theory study on work related stress in professionals who provide health & social care for people who exhibit behaviours that challenge
Source: PLoS One. 2020 Feb 27;15(2):e0229706. doi: 10.1371/journal.pone.0229706 (PMC7046268; doi:10.1371/journal.pone.0229706)
Supplement: S1 File — (DOCX) [file pone.0229706.s001.docx]

| **Professional Group** | **Method of Data Collection** | **n** | **Participant Demographics** |
| --- | --- | --- | --- |
| Community Mental Healthcare Team | Focus Group | 10 | Participant 1 was a Service Manager, male, aged 56 years and with 108 months of experience in their role.  Participant 2 was a Support Worker, male, aged 31 years and with 36 months of experience in their role  Participant 3 was a Support Worker, male, aged 56 years and with 24 months of experience in their role.  Participant 4 was an Assistant Service Manager, female, aged 40 years and with 96 months of experience in their role.  Participant 5 was a Senior Support Worker, female, aged 60 years and with 132 months of experience in their role.  Participant 6 was a Housing Support Worker, female, aged 40 years and with 3 months experience in their role.  Participant 7 was a Support Worker, female, aged 41 and with 15 months experience in their role.  Participant 8 was a Housing Support Worker, female, aged 34 and with 3 months experience in their role.  Participant 9 was a Senior Support Worker, male, aged 64 years and with 156 months of experience in their role.  Participant 10 was a Senior Support Worker, female, aged 48 years and with 120 months of experience in their role. |
| Children and Younger People’s Mental Health Inpatient Setting | 1:1 Semi-structured interview | 1 | Participant 11 was a Staff Nurse, female, aged 53 years and with 45 months of experience in their role. |
| Learning Disability Inpatient Setting | 1:1 Semi-structured interview | 1 | Participant 12 was a Staff Nurse, female, aged 27 years and with 21 months of experience in their role. |
| Children and Younger People’s Mental Health Inpatient Setting | 1:1 Semi-structured interview | 1 | Participant 13 was a Support Worker, female, aged 20 and with 20 months of experience in their role. |
