# Supplementary material for: A grounded theory study on work related stress in professionals who provide health & social care for people who exhibit behaviours that challenge
Source: PLoS One. 2020 Feb 27;15(2):e0229706. doi: 10.1371/journal.pone.0229706 (PMC7046268; doi:10.1371/journal.pone.0229706)
Supplement: S2 File — (DOCX) [file pone.0229706.s002.docx]

| **Professional Group** | **Method of Data Collection** | **n** | **Participant Demographics** |
| --- | --- | --- | --- |
| Community and Residential Autism Services | Focus Group | 4 | Participant 14 was a Support Worker, female, aged 22 years and with 10 months of experience in their role.  Participant 15 was a Support Worker, female, aged 63 years and with 168 months of experience in their role  Participant 16 was a Support Worker, female, aged 48 years and with 101 months of experience in their role.  Participant 17 was a Senior Support Worker, female, aged 47 years and with 166 months of experience in their role. |
| Community and Residential Autism Services | Focus Group | 5 | Participant 18 was a Support Worker, female, aged 43 years and with 65 months of experience in their role.  Participant 19 was a Support Worker, female, aged 40 years and with 68 months of experience in their role.  Participant 20 was a Senior Support Worker, female, aged 32 years and with 48 months of experience in their role.  Participant 21 was a Support Worker, female, aged 24 years and with 12 months of experience in their role.  Participant 22 was a Support Worker, female, aged 42 years and with 190 months of experience in their role. |
| Community Mental Health Team | Focus Group | 3 | Participant 23 was a Clinical Psychologist, male, aged 38 years and with 65 months of experience in their role.  Participant 24 was a Challenging Behaviour Nurse, female, aged 53 years and with 24 months of experience in their role.  Participant 25 was a Directorate Manager for Community Services, female, aged 49 years and with 48 months of experience in their role. |
| Autism Community Service | Focus Group | 5 | Participant 26 was a Support Worker, male, aged 24 and with 3 months of experience in their role.  Participant 27 was a Support Worker, female, aged 45 years and with 3 months of experience in their role.  Participant 28 was a Support Worker, female, aged 25 years and with 3 months of experience in their role.  Participant 29 was a Support Worker, female, aged 26 years and with 3 months of experience in their role.  Participant 30 was a Support Worker, female, aged 28 years and with 3 months of experience in their role. |
| Learning Disability Residential Service |  | 5 | Participant 31 was a Clinical Nurse Specialist, male, aged 46 years and with 96 months of experience in their role.  Participant 32 was a Support Worker, male, aged 24 years and with 15 months experience in their role.  Participant 33 was a Support Worker, female, aged 24 years and with 15 months of experience in their role.  Participant 34 was an Assistant Psychologist, male, aged 28 years, and with 18 months of experience in their role.  Participant 35 was a Staff Training Officer, male, aged 52 years and with 60 months of experience in their role. |
| Community Autism Service |  | 5 | Participant 36 was a Team Leader, male, aged 38 years and with 3 years 11 months of experience in their role.  Participant 37 was a Support Worker, male, aged 25 years and with 4 years 1 month of experience in their role.  Participant 38 was a Team Leader, female, aged 53 years and with 60 months of experience in their role.  Participant 39 was a Team Leader, female, aged 50 years and with 48 months of experience in their role.  Participant 40 was a Support Worker, female, aged 21 years and with 11 months of experience in their role. |
| Drug and Alcohol Rehabilitation Service |  | 5 | Participant 41 was a Needle Exchange Assistant, aged 45 years and with 15 months of experience in their role.  Participant 42 was a Community Clinical Manager, female, aged 28 years and 2 months experience in their role.  Participant 43 was a Drug Rehabilitation Lead, male, aged 61 years and with 112 months experience in their role.  Participant 44 was a Duty Worker, female, aged 54 years and with 36 months of experience in their role.  Participant 45 was a Clinical Lead, female, aged 32 years and with 1 month of experience in their role. |
| Organic Inpatient and Older Adult Community Service | 1:1 Semi-structured interview | 1 | Participant 46 was a Registered Mental Health Nurse, aged 34 years and 54 months experience in their role. |
| Autism Community and Residential Service | 1:1 Semi-structured interview | 1 | Participant 47 was a Behaviour Nurse Specialist, male, aged 53 years and with 120 months of experience in their role. |
| Community Mental Healthcare Team | 1:1 Semi-structured interview | 1 | Participant 6 was a Housing Support Worker, female, aged 40 years and with 12 months experience in their role. |
